# The predictive performance of artificial intelligence on the outcome of stroke: a systematic review and meta-analysis

**DOI:** 10.3389/fnins.2023.1256592

**Published:** 2023-09-07

**Authors:** Yujia Yang, Li Tang, Yiting Deng, Xuzi Li, Anling Luo, Zhao Zhang, Li He, Cairong Zhu, Muke Zhou

**Affiliations:** ^1^Department of Neurology, West China Hospital, Sichuan University, Chengdu, Sichuan, China; ^2^Department of Epidemiology and Health Statistics, West China School of Public Health and West China Fourth Hospital, Sichuan University, Chengdu, Sichuan, China

**Keywords:** artificial intelligence, machine learning, deep learning, acute stroke, outcome, prognosis, prediction

## Abstract

**Objectives:**

This study aimed to assess the accuracy of artificial intelligence (AI) models in predicting the prognosis of stroke.

**Methods:**

We searched PubMed, Embase, and Web of Science databases to identify studies using AI for acute stroke prognosis prediction from the database inception to February 2023. Selected studies were designed cohorts and had complete data. We used the Quality Assessment of Diagnostic Accuracy Studies tool to assess the qualities and bias of included studies and used a random-effects model to summarize and analyze the data. We used the area under curve (AUC) as an indicator of the predictive accuracy of AI models.

**Results:**

We retrieved a total of 1,241 publications and finally included seven studies. There was a low risk of bias and no significant heterogeneity in the final seven studies. The total pooled AUC under the fixed-effects model was 0.872 with a 95% CI of (0.862–0.881). The DL subgroup showed its AUC of 0.888 (95%CI 0.872–0.904). The LR subgroup showed its AUC 0.852 (95%CI 0.835–0.869). The RF subgroup showed its AUC 0.863 (95%CI 0.845–0.882). The SVM subgroup showed its AUC 0.905 (95%CI 0.857–0.952). The Xgboost subgroup showed its AUC 0.905 (95%CI 0.805–1.000).

**Conclusion:**

The accuracy of AI models in predicting the outcomes of ischemic stroke is good from our study. It could be an assisting tool for physicians in judging the outcomes of stroke patients. With the update of AI algorithms and the use of big data, further AI predictive models will perform better.

## 1. Background

Artificial intelligence (AI) can be defined as the ability of computers or other machines to demonstrate or simulate intelligent behavior, like human beings (Krittanawong et al., 2017; Garcia-Vidal et al., 2019; Schwalbe and Wahl, 2020; Bonkhoff and Grefkes, 2022). Machine learning (ML) is one way to implement AI, which has shown the greatest potential in dealing with problems involving unstructured data, such as image recognition (Deo, 2015; Esteva et al., 2019). ML techniques utilize various methods for automated data analysis, including logistic regression (LR), random forests (RF), support vector machines (SVM), and classification trees, which allow combining features (data characteristics) with flexible decision boundaries in a non-linear manner. The advent of neural networks (NN) and deep learning (DL) techniques has changed the ML domain and achieved automatic and efficient feature recognition and processing in covert analysis networks without prior feature selection. There were some studies suggesting that ML and DL have again recently achieved substantial improvements and demonstrated comparable performance to trained physicians in the fields of other departments, like radiology and dermatology (Gulshan et al., 2016; Esteva et al., 2017; Hannun et al., 2019).

Acute stroke ranks among the leading causes of morbidity and mortality worldwide, and it can be divided into ischemic stroke and hemorrhagic stroke (Toyoda et al., 2022). In addition, predicting the outcome of a stroke often depends on the experience of the physician clinically, but it is difficult for inexperienced young physicians to judge the prognosis. In clinical, patients are most concerned about their clinical outcomes. Imagine that you are a young inexperienced physician and you are on duty in the ward, your patient asks you about the outcomes after treatment and you cannot ensure the judgment is right based on your own experience. If there is an objective tool at hand to predict the prognosis according to the patient’s condition quickly and accurately, using this tool to corroborate your judgment will make you more confident in judging the prognosis of your patient. Exactly, AI predictive models can bring objective results after learning input features and countless calculations. ML predictive models which are image-based feature recognition and segmentation and have greatly facilitated the rapid diagnosis of stroke, but stroke prognosis depends on a large number of patient-specific and clinical factors, so accurate prognostic prediction models remain challenging (Mendelson and Prabhakaran, 2021; Toyoda et al., 2022).

Although previous studies on predicting stroke prognosis also used many AI algorithms, the overall accuracy of AI models in predicting stroke prognosis is inconsistent. Tree-based algorithms own favorable interpretability and a relatively simple algorithm, and researchers that used RF algorithms performed high-accuracy prediction prognosis of acute ischemic stroke patients (AUC = 0.936 ± 0.034) and primary intracerebral hemorrhage stroke patients (AUC = 0.917) (Monteiro et al., 2018; Wang et al., 2019). However, the samples of these studies were not “big” datasets and had relatively poor representativeness. SVM is frequently used in predicting stroke outcomes relying on neuroimaging data and showing moderate to high accuracy prediction of prognosis, with an AUC ranging from 0.788 to 0.92 (Forkert et al., 2015; Giacalone et al., 2018; Lin et al., 2018; Nishi et al., 2019; Li et al., 2022; Roh et al., 2022). The present most complex algorithms, deep neural network (DNN), a model of DL, ran a bigger sample analysis and performed high accuracy prediction prognosis (AUC = 0.904) in minor stroke patients (Sung et al., 2020) and moderate accuracy prediction prognosis (AUC = 0.88 ± 0.12 and 0.888 ± 0.008) in acute ischemic stroke (Nielsen et al., 2018; Heo et al., 2019) but had poor interpretability for input variables.

This study aimed to analyze the literature to explore the accuracy of AI models in stroke outcome prediction and compare the AUC among different algorithms.

## 2. Methods

We performed this study according to the Preferred Reporting Items for Systematic Reviews and Meta-Analyses (PRISMA) guidelines (McInnes et al., 2018).

### 2.1. Selection criteria

**Figure 1:**
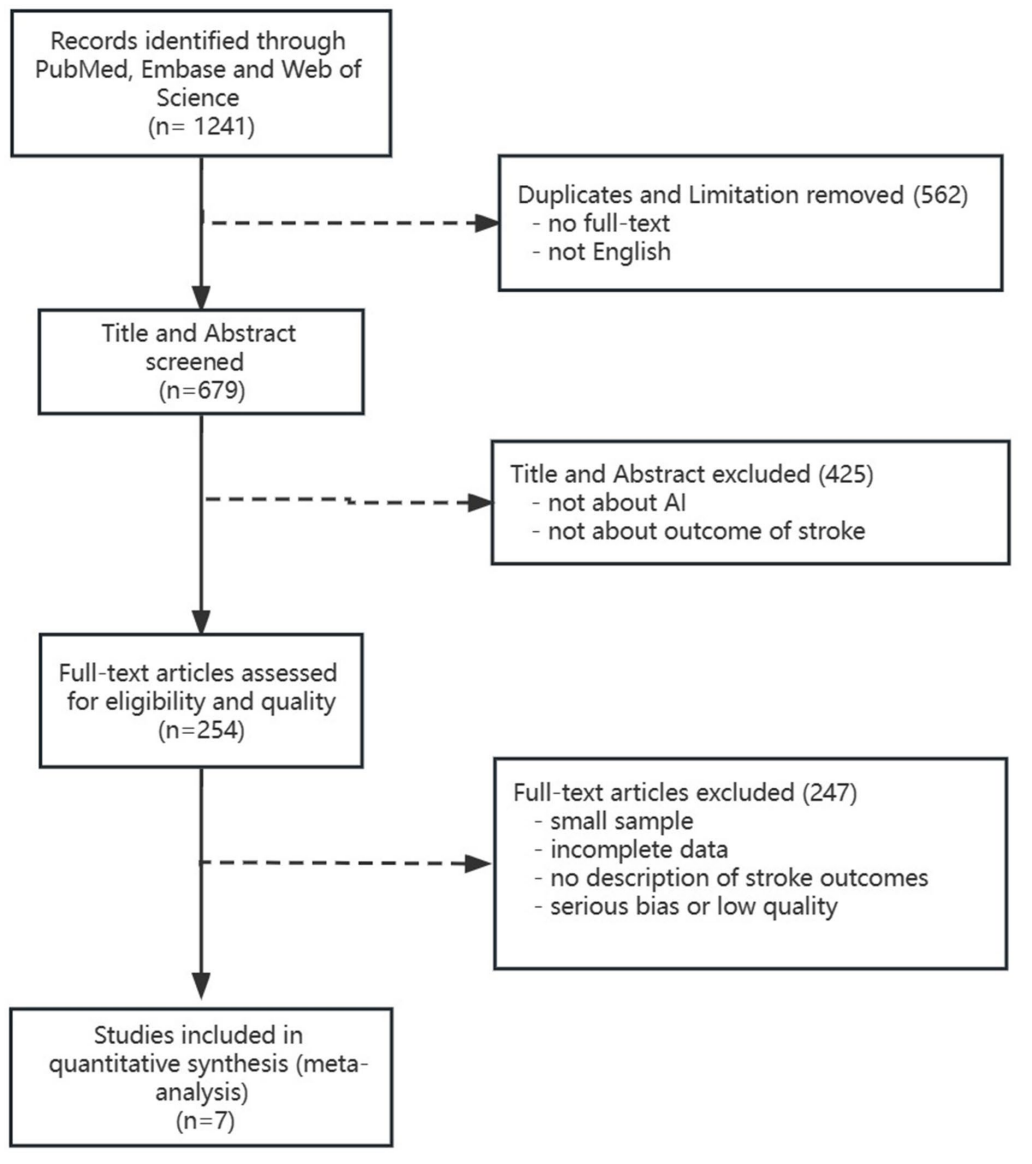
Flow chart of included literature.

We searched “*acute stroke*” “*artificial intelligence*” “*deep learning*” “*machine learning*” “*prognosis*” and “*outcome*” in PubMed, Embase, and Web of Science databases from the inception to February 2023 and assessed eligible articles by screening titles and abstracts followed by full-text evaluation (Figure 1). In addition, we formulated our included studies as follows: (1) Population: patients diagnosed with acute ischemic or hemorrhagic stroke in retrospective and prospective cohorts and had prognosis data. The prognosis of stroke not only included the functional outcomes but also the radiological outcomes, the likelihood of morbidity, and mortality (Toyoda et al., 2022). (2) Index test: the predictive prognosis results of AI. (3) Reference standard: recognized prognosis recorded in included studies. (4) Outcomes: area under the curve (AUC) with its 95% confidence interval or standard error of receiver operator characteristic in AI models on stroke prognosis.

### 2.2. Data extraction and quality assessment

Two independent investigators extracted the following information from the included studies: first author, publication year, country, population data (age and sex), and outcomes. Data extraction forms included details on the included study characteristics. Two investigators assessed the quality and bias of studies independently by using The Revised Tool for the Quality Assessment of Diagnostic Accuracy Studies (QUADAS) scores. In the whole process of data extraction and quality assessment, all different opinions were solved through discussion with the third reviewer.

### 2.3. Statistical analysis

We pooled the outcome data using a fixed effects model. Heterogeneity among studies was evaluated using the *Q* statistic and *I*-squared test (*I*^2^). Significant heterogeneity was defined as *p*-value <0.05 or *I*^2^ > 50%. For sensitivity analyses, one by one elimination method was performed to investigate the robustness of the results. All statistical analyses were performed using MedCalc® statistical software version 22.009 (MedCalc Software Ltd., Ostend, Belgium^1^; 2023).

## 3. Results

### 3.1. Search results and included studies characteristics

We retrieved a total of 1,241 publications. Finally, we included seven studies with 4,379 ischemic stroke participants. The detailed flow chart is shown in Figure 1. In the seven included studies, there were 17 predictive models and they were divided into five subgroups according to their algorithms (SVM, RF, LR, DL, and Xgboost).

The characteristics of all included studies are shown in Table 1. In our review, four articles (Monteiro et al., 2018; Heo et al., 2019; Nishi et al., 2019; Li et al., 2022) used mRS as functional outcomes, and they all thought mRS ≤ 2 was a good outcome. The other three articles (Lin et al., 2018; Nielsen et al., 2018; Grosser et al., 2020) used radiological biomarkers, follow-up lesion volume, and neurological deterioration as outcomes.

**Table 1:** The characteristics of included studies.

First author, publication year	Country	Population	Sample size，*n*	AI-based algorithm	AUC
Heo et al. (2019)	Korea	AIS patients	2604	DNN	0.888
				RF	0.857
				LR	0.849
Nielsen et al. (2018)	Denmark	AIS patients	222	CNN_deep_	0.88
Lin et al. (2018)	China	AIS patients	382	SVM	0.849
Li et al. (2022)	China	Stroke patients	260	SVM	0.92
Nishi et al. (2019)	Japan	AIS patients with LVO	387	SVM	0.86
				RLR	0.86
				RF	0.85
Monteiro et al. (2018)	Portugal	AIS patients	425	RF	0.936
				LR	0.926
				SVM	0.909
				Decision Tree	0.916
				Xgboost	0.911
Grosser et al. (2020)	Germany	Anterior circulation strokes patients	99	Xgboost	0.893
				LR	0.877
				RF	0.891

AIS, Acute ischemic stroke. DNN, Deep neural network. CNN_deep_, Deep convolutional neural network. SVM, Support vector machine. RF, Random Forest. RLR, Regularized logistic regression. LVO, Large vessel occlusion.

### 3.2. Quality assessment

**Figure 2:**
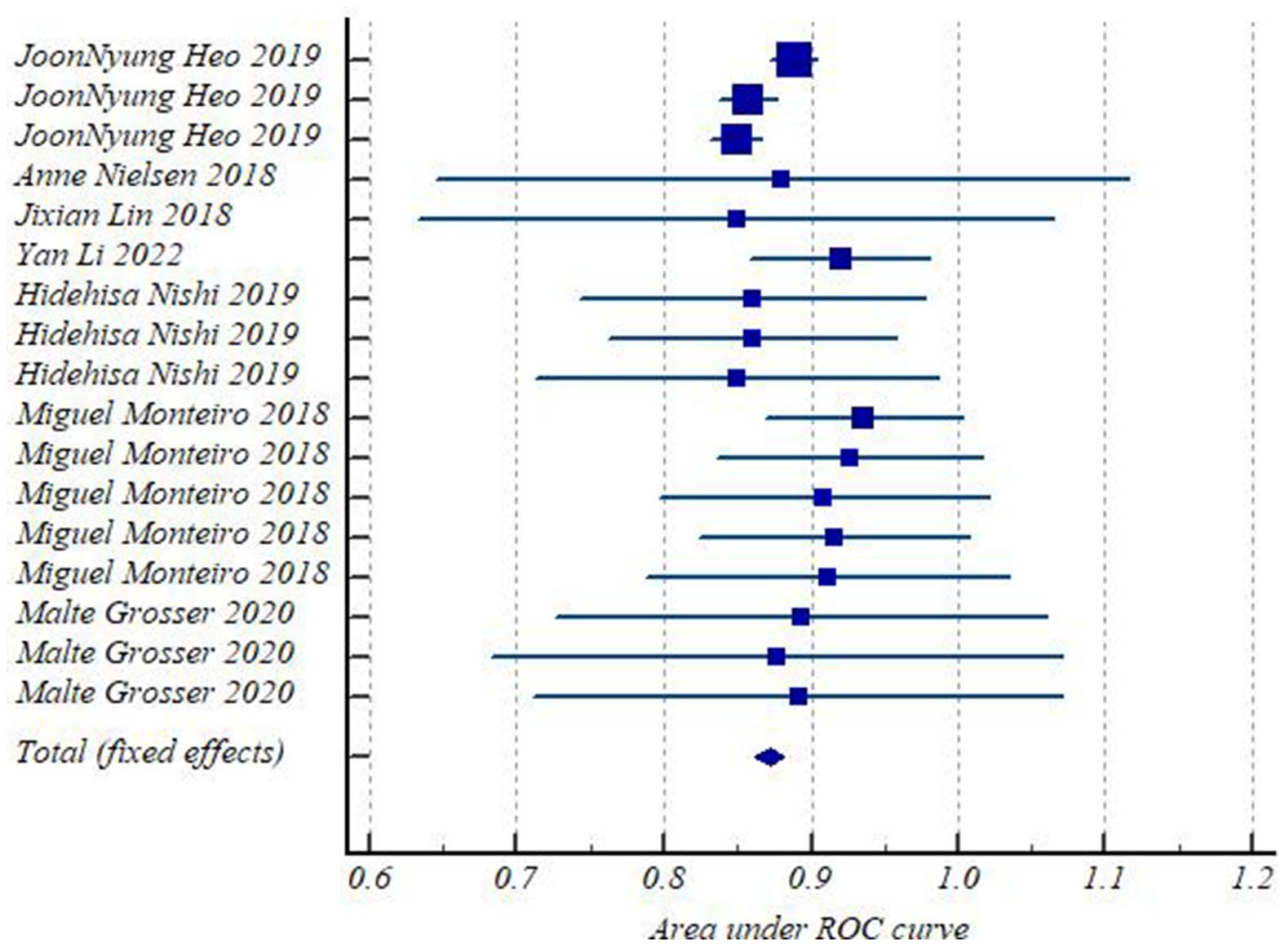
Forest plots of included studies.

All included studies had low to moderate risks in QUADAS scores, and the risk of bias is shown in Figure 2. The heterogeneity test of studies for analyzing AUC showed *I*^2^ = 27.67%, suggesting that there was no significant heterogeneity in the study.

### 3.3. The AUC of included studies

**Figure 3:**
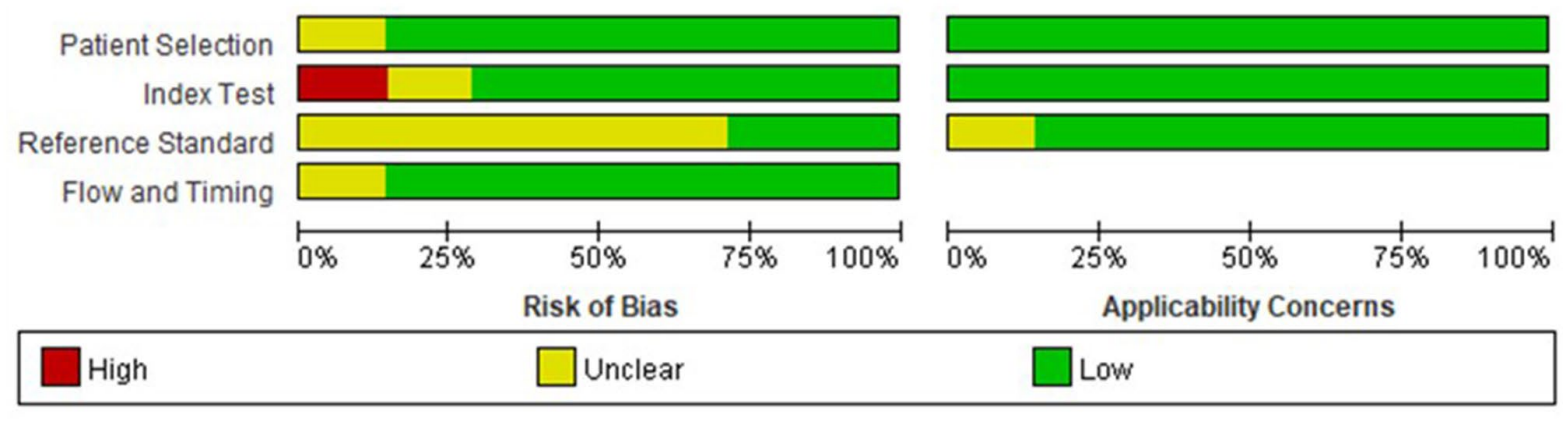
The risk of bias in the included studies.

Forest plots (Figure 3) presented AUC and its 95% CI for the included 17 models in turn. The pooled AUC under the fixed-effects model was 0.872 with a 95% CI of (0.862–0.881) (Table 2).

**Table 2:** The total included AUC under the random-effects model.

Study	Algorithm	ROC area	Standard error	95% CI	*p*	Weight (%)
Heo et al. (2019)	DNN	0.888	0.00800	0.872–0.904		36.55
	RF	0.857	0.0100	0.837–0.877		23.39
	LR	0.849	0.00900	0.831–0.867		28.88
Nielsen et al. (2018)	CNN_deep_	0.880	0.120	0.645–1.000		0.16
Lin et al. (2018)	SVM	0.849	0.110	0.633–1.000		0.19
Li et al. (2022)	SVM	0.920	0.0310	0.859–0.981		2.43
Nishi et al. (2019)	SVM	0.860	0.0600	0.742–0.978		0.65
	RLR	0.860	0.0500	0.762–0.958		0.94
	RF	0.850	0.0700	0.713–0.987		0.48
Monteiro et al. (2018)	RF	0.936	0.0340	0.869–1.000		2.02
	LR	0.926	0.0460	0.836–1.000		1.11
	SVM	0.909	0.0570	0.797–1.000		0.72
	Decision Tree	0.916	0.0470	0.824–1.000		1.06
	Xgboost	0.911	0.0630	0.788–1.000		0.59
Grosser et al. (2020)	Xgboost	0.893	0.0850	0.726–1.000		0.32
	LR	0.877	0.0990	0.683–1.000		0.24
	RF	0.891	0.0920	0.711–1.000		0.28
Total (fixed effects)		0.872	0.00484	0.862–0.881	<0.001	100.00

Table 3 shows the subgroup results of this meta-analysis. The DL subgroup showed an AUC of 0.888 (95%CI 0.872–0.904). The LR subgroup showed an AUC of 0.852 (95%CI 0.835–0.869). The RF subgroup showed an AUC of 0.863 (95%CI 0.845–0.882). The SVM subgroup showed an AUC of 0.905 (95%CI 0.857–0.952). The Xgboost subgroup showed an AUC of 0.905 (95%CI 0.805–1.000). All results showed a good performance of AI models in predicting the outcome of stroke patients (Mandrekar, 2010).

**Table 3:** The subgroup analysis of pooled AUC.

Algorithm	Study	AUC	Standard error	95% CI
DL	Heo et al. (2019)	0.888	0.008	0.872–0.904
	Nielsen et al. (2018)	0.880	0.120	0.645–1.000
	AUC	0.888	0.00798	0.872–0.904
LR	Heo et al. (2019)	0.849	0.009	0.831–0.867
	Nishi et al. (2019)	0.860	0.050	0.762–0.958
	Monteiro et al. (2018)	0.926	0.046	0.836–1.000
	Grosser et al. (2020)	0.877	0.099	0.683–1.000
	Total	0.852	0.00866	0.835–0.869
RF	Heo et al. (2019)	0.857	0.010	0.837–0.877
	Nishi et al. (2019)	0.850	0.070	0.713–0.987
	Monteiro et al. (2018)	0.936	0.034	0.869–1.000
	Grosser et al. (2020)	0.891	0.092	0.711–1.000
	Total	0.863	0.00945	0.845–0.882
SVM	Lin et al. (2018)	0.849	0.110	0.633–1.000
	Li et al. (2022)	0.920	0.031	0.859–0.981
	Nishi et al. (2019)	0.860	0.060	0.742–0.978
	Monteiro et al. (2018)	0.909	0.057	0.797–1.000
	Total	0.905	0.0242	0.857–0.952
Xgboost	Monteiro et al. (2018)	0.911	0.063	0.788–1.000
	Grosser et al. (2020)	0.893	0.085	0.726–1.000
	Total	0.905	0.0506	0.805–1.000

## 4. Discussion

To our knowledge, this is the first meta-analysis to study AI models’ performance in predicting stroke outcomes. In the final included studies, the participants were ischemic stroke patients. Many studies on the outcome prediction of hemorrhagic stroke did not meet our inclusion criteria. Thus, our results apply to ischemic stroke patients only. According to our results, the overall performance of the predictive model for ischemic stroke outcomes is good. In subgroup analysis, the SVM was the most accurate and the LR models were the least in terms of comparison AUC.

Previously, many studies in stroke outcomes used some variant of linear regression models, which are generally easier to interpret, but they do not automatically exploit nonlinear relationships and interactions, leading to poor prediction accuracy. The prognostic models from the Virtual International Stroke Trials Archive on functional outcome and survival are AUC 0.808 and 0.706 (Konig et al., 2008), respectively, which is a relatively accurate and simple prediction scale, but compared to AI predictive models, AI models show better accurate performance. The biomarker-based CoRisk score was AUC 0.819, of which the score components were copeptin levels, age, NIH Stroke Scale, and recanalization therapy (De Marchis et al., 2019). The prediction model score showed relatively good accuracy and interpretation, but whether it is necessary to detect the plasma copeptin levels in clinical is still to be decided. In our study, the participants that included in our analysis used rapid prediction from medical data obtained at the time of presentation at the emergency department or obtained imaging data after admission or interventional procedures. This indicates that the use of AI to build a prediction model has better clinical applicability. However, clinicians typically have a limited understanding of this methodology. Therefore, we conducted this systematic review and meta-analysis to clarify how AI models can provide stroke outcome prediction.

In addition, the points of prediction tools in clinical practice are feasibility and acceptability as overly complex scales will not be used in acute stroke. Although DL models have good predictive accuracy, the complexity and non-interpretability of models limit their clinical application. According to our results, though SVM and Xgboost models had similar AUC, SVM seemed to perform better than Xgboost in predicting stroke outcomes due to discrete linear data or proper nonlinear kernels that fit the data better by improved generalization (Noble, 2006; Roh et al., 2022). Regrettably, we did not know why researchers chose the specific algorithm for their predictive models during the literature review.

In contrast to traditional predictive scores, most AI predictive models share a common set of independent demographic variables, laboratory values, and imaging feathers. While some variables and characteristics are not well validated individually in clinical, they may add predictive value in some cases. A comparative study of ML algorithms and traditional risk models is needed. If these studies demonstrate the advantages of ML-based prediction, then optimization algorithms can be implemented through electronic health records to facilitate the application of clinical practice.

The limitations of our study are that we assessed the accuracy of the prediction only through the AUC, not exactly focusing on the sensitivity, specificity, and accuracy rate because of incomplete data for meta-analysis. We decided to use AUC to assess the accuracy of predictive models because AUC could remain stable even when the distribution of positive and negative samples in the test set changed. Therefore, despite the simplicity of our results, they were explanatory and reliable. What is more? Because of the large scope of our review, our goal was systematic rather than comprehensive. Therefore, we might miss some relevant studies, but we consider it unlikely that these studies were of higher quality than those already included. Another problem was the sample size included in the literature, none of which could be called as big data; only one study of more than 1,000 participants, and according to previous studies, there are a large number of candidate predictor variables to analyze, so future AI predictive models must be developed for a large number of patients.

## 5. Conclusion

AI predictive models have high accuracy in predicting the outcome of stroke, which assists physicians to judge the specific outcome of a patient and adjust the treatment plan according to the outcome of the judgment.

## Data availability statement

The original contributions presented in the study are included in the article/supplementary material, further inquiries can be directed to the corresponding authors.

## Author contributions

YY: Conceptualization, Data curation, Formal analysis, Methodology, Software, Writing – original draft, Writing – review & editing. LT: Investigation, Methodology, Writing – review & editing. YD: Data curation, Writing – review & editing. XL: Writing – review & editing. AL: Writing – review & editing, Data curation. ZZ: Writing – review & editing. LH: review & editing. CZ: Methodology, Writing – review & editing. MZ: Funding acquisition, Methodology, Project administration, Writing – review & editing.

## Funding

The author(s) declare financial support was received for the research, authorship, and/or publication of this article. The China National Key Research and Development Project, 2021YFC2500506, supported this work.

## Conflict of interest

The authors declare that the research was conducted in the absence of any commercial or financial relationships that could be construed as a potential conflict of interest.

## Publisher’s note

All claims expressed in this article are solely those of the authors and do not necessarily represent those of their affiliated organizations, or those of the publisher, the editors and the reviewers. Any product that may be evaluated in this article, or claim that may be made by its manufacturer, is not guaranteed or endorsed by the publisher.

## Footnotes

^1^https://www.medcalc.org

## Abbreviations

AI: Artificial intelligence
ML: Machine learning
DL: Deep learning
mRS: modified Rankin Scale
AUC: area under curve
RF: random forest
SVM: support vector machine
LR: Logistic regression
